# Functional Vitamin B12 Deficiency Induced by Nitrous Oxide: Case Report of Acute Psychosis and Severe Neuropathy

**DOI:** 10.1192/bjo.2026.11766

**Published:** 2026-06-30

**Authors:** Rishabh Bajaj, Ajay Baskaran, Asma Javed

**Affiliations:** Black Country Healthcare NHS Foundation Trust, Black Country, United Kingdom

## Abstract

**Aims:**

Recreational nitrous oxide use is increasing and widely seen as low risk, but chronic exposure can inactivate vitamin B12, causing deficiency even with normal serum levels. This may lead to psychiatric, neurological, and haematological symptoms, complicating diagnosis for mental health professionals.

**Methods:**

A middle-aged man with no psychiatric history, was brought to the hospital due to confusion, reduced awareness, and incoherent speech. He exhibited marked paranoia, believing others intended harm, and would only trust his mother for basic needs. He expressed a fear of losing consciousness and believed he needed to be “shocked”. During admission, his behaviour escalated to aggression, including assaulting staff and breaking a window. He also experienced multiple episodes of loss of consciousness.

Initial investigations revealed severe anaemia, leukopenia, raised inflammatory markers, and low serum vitamin B12. Further diagnostic work-up, including MRI of the head, lumbar puncture, abdominal ultrasound, and comprehensive infective, autoimmune, paraneoplastic, and toxicology screens, were unremarkable. Psychosocial evaluation uncovered recent significant stressors, including multiple bereavements and a history of imprisonment. Importantly, the patient disclosed heavy nitrous oxide use–6 to 10 canisters daily for four months–and past cannabis use, though he denied using cannabis recently.

He was treated for an infection and commenced on intramuscular hydroxocobalamin (vitamin B12), which improved his mental state and restored his decision-making abilities. However, he self-discharged after a week of treatment, against medical advice.

Within days, the patient returned with rapidly worsening lower-limb weakness, numbness, tingling, impaired balance, and recurrent falls. Physical examination revealed reduced power in the distal lower limbs (grade 3–4/5), absent ankle reflexes, impaired vibration and proprioception, and a broad-based, ataxic gait. Nerve conduction studies demonstrated severe motor axonal neuropathy. These findings were consistent with functional vitamin B12 deficiency due to nitrous oxide use, despite near-normal serum B12 levels.

**Results:**

Nitrous oxide can cause functional vitamin B12 deficiency by oxidizing its cobalt ion, impairing methionine synthase and myelin synthesis–even when serum B12 appears normal. This complicates diagnosis and may present as psychiatric symptoms like psychosis or delirium, highlighting the importance of collaboration between psychiatry and general medicine. Early identification and parenteral B12 therapy are crucial to prevent lasting neurological damage.

**Conclusion:**

Clinicians should consider nitrous oxide toxicity in patients with unexplained neuropsychiatric or haematological symptoms, especially if there is a relevant use history. Prompt recognition and treatment of functional vitamin B12 deficiency are vital to prevent serious neurological complications.

